# Machine Learning-Based Classification to Disentangle EEG Responses to TMS and Auditory Input

**DOI:** 10.3390/brainsci13060866

**Published:** 2023-05-27

**Authors:** Andrea Cristofari, Marianna De Santis, Stefano Lucidi, John Rothwell, Elias P. Casula, Lorenzo Rocchi

**Affiliations:** 1 Department of Civil Engineering and Computer Science Engineering, “Tor Vergata” University of Rome, 00133 Rome, Italy; andrea.cristofari@uniroma2.it; 2 Department of Computer, Automatic and Management Engineering, “Sapienza” University of Rome, 00185 Rome, Italy; marianna.desantis@uniroma1.it (M.D.S.); lucidi@diag.uniroma1.it (S.L.); 3 Department of Clinical and Movement Neurosciences, UCL Queen Square Institute of Neurology, University College London, London WC1N 3BG, UK; j.rothwell@ucl.ac.uk; 4 Department of System Medicine, “Tor Vergata” University of Rome, 00133 Rome, Italy; elias.casula@gmail.com; 5 Department of Medical Sciences and Public Health, University of Cagliari, Cittadella Universitaria di Monserrato, 09042 Cagliari, Italy

**Keywords:** transcranial magnetic stimulation, electroencephalography, TMS-EEG, evoked potentials, machine learning, neural networks

## Abstract

The combination of transcranial magnetic stimulation (TMS) and electroencephalography (EEG) offers an unparalleled opportunity to study cortical physiology by characterizing brain electrical responses to external perturbation, called transcranial-evoked potentials (TEPs). Although these reflect cortical post-synaptic potentials, they can be contaminated by auditory evoked potentials (AEPs) due to the TMS click, which partly show a similar spatial and temporal scalp distribution. Therefore, TEPs and AEPs can be difficult to disentangle by common statistical methods, especially in conditions of suboptimal AEP suppression. In this work, we explored the ability of machine learning algorithms to distinguish TEPs recorded with masking of the TMS click, AEPs and non-masked TEPs in a sample of healthy subjects. Overall, our classifier provided reliable results at the single-subject level, even for signals where differences were not shown in previous works. Classification accuracy (CA) was lower at the group level, when different subjects were used for training and test phases, and when three stimulation conditions instead of two were compared. Lastly, CA was higher when average, rather than single-trial TEPs, were used. In conclusion, this proof-of-concept study proposes machine learning as a promising tool to separate pure TEPs from those contaminated by sensory input.

## 1. Introduction

The combination of transcranial magnetic stimulation (TMS) and electroencephalography (EEG) has become an increasingly used approach to assess cortical physiology in healthy humans [1,2,3,4] and patients affected by disorders of the central nervous system [5,6,7]. There is considerable evidence to support the notion that EEG signals following TMS, either measured as transcranial-evoked potentials (TEPs) or oscillations, mostly reflect the summation of excitatory and inhibitory post-synaptic potentials generated by direct activation of cortical neurons [8,9,10]. However, recent research has demonstrated that auditory and somatosensory input caused by TMS may give rise to evoked responses which can contaminate the TEP [11,12,13]. This is particularly the case for the TMS click, which can result in auditory-evoked potentials (AEPs), mostly consisting in a negative/positive complex (N100/P200) distributed at the vertex, compatible with saliency-related multimodal responses [13,14].

Whereas differences between TEPs and AEPs are clear in early (<70 ms) and late (>120 ms) signals [13], they might be more subtle around 100 ms. The N100 of the N100/P200 AEP complex (AEP-N100) has recently been demonstrated to be physiologically distinct from a similar N100 (TEP-N100) wave, which is part of the TEP obtained by stimulation of the primary motor cortex (M1) [3,15]. In addition to its central location and to the fact that it is invariably followed by a P200, the AEP-N100 differs from the TEP-N100 by its shorter latency and suppression by appropriate countermeasures (i.e., the use of noise masking and/or ear defenders) [13,16,17]. By contrast, the TEP-N100 persists even after optimal suppression of the TMS click [5,13], is located at the stimulation site [3,15], has a longer latency, and is not followed by a P200 [13] (Table 1).

Despite the aforementioned differences, the spatial and temporal distribution of the two N100 might be similar enough to prevent accurate discrimination by common statistical approaches used in the TMS-EEG field [13]. Contamination of TEPs by AEPs might be even greater in case of suboptimal masking of the TMS click. This might occur for several reasons, including high TMS intensity, low tolerance for the masking noise by test subjects, or experimental settings which entail particularly loud stimulation, such as cerebellar TMS [18,19]. Therefore, efficient computational approaches to discriminate TEPs from AEPs and, by extension, to understand whether the former are contaminated by the latter, are highly desirable.

In recent years, machine learning algorithms have been widely used in different fields, such as engineering, economics, biology, and medicine [20]. The success of these algorithms lies in their ability to identify relationships among data which are otherwise hard to reveal. In particular, these methods try to approximate unknown laws that rule a complex real-world system by using samples observed from the system itself. When dealing with classification problems, the system is a model that associates a given input to a certain group or class. The goal is to build a “machine” able to correctly classify any input. This mechanism of exploiting observed data is called “learning”, which is traditionally divided into supervised and unsupervised. In supervised learning, the machine is “trained” on samples that are input–output pairs, where the input is the set of sample features, while the output is the group to which the sample belongs. A solution to address classification problems is to use the so-called artificial neural networks, representing one of the most common tools in supervised learning. As the name suggests, these networks try to simulate the highly connected brain neural system and are made of one or more layers of parallel “neurons” that receive an input and produce an output. In particular, the input of each neuron is the weighted sum of the outputs of the neurons of the previous layer, passing through an activation function. The input of each neuron is typically transformed through a sigmoidal function into a scalar value that can be interpreted as a Yes/No output of the neuron. In classification problems, the input to the first layer is a weighted sum of the sample features, while the output from the last layer represents the class predicted by the artificial neural network. Roughly speaking, the training process consists of choosing the weights that minimize a measure of the error between the true and the predicted classifications. When the groups to which the samples belong are unknown, unsupervised learning techniques can be used to find out some patterns in the data. Relevant examples include the clustering and community detection problems, where the aim is to divide data into groups based on some measure of similarity or on the density of the connections [21,22,23].

The aforementioned features make machine learning algorithms particularly suited to discriminate data based on subtle differences. Additionally, such computational solutions are critically helpful when generating predictions with large and unwieldy data, where the number of input variables far exceeds the number of subjects [24], such as TMS-EEG signals. Therefore, we tested machine learning ability to differentiate TEPs recorded with noise masking, TEPs without masking, and AEPs, in conditions where common statistical approaches provided suboptimal results [13].

## 2. Materials and Methods

**Figure 1:**
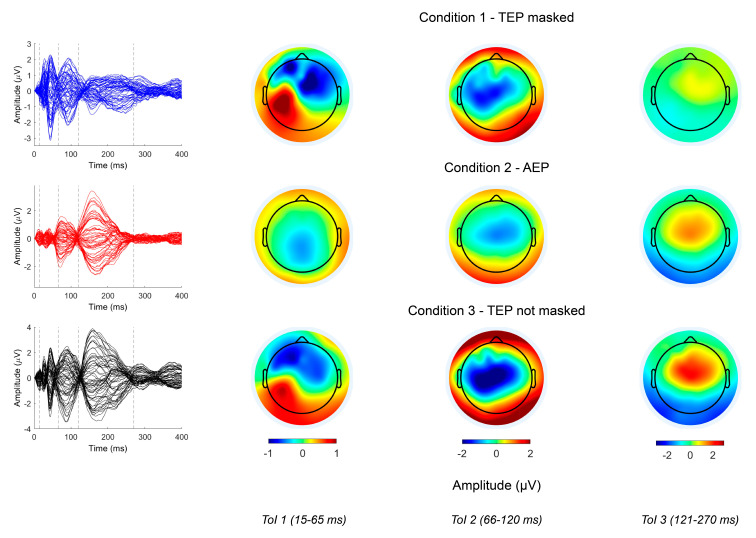
Butterfly plots (left row) and topographical plots of TEPs in the early, middle, and late ToIs, and three different stimulation conditions (C1–TEP masked, upper row; C2–AEP, middle row; C3–TEP not masked, lower row).

The aim of the present set of experiments was to classify TEPs obtained in different conditions with regards to contamination of AEPs and pure auditory responses (Figure 1).

The data were included in a previously published work, to which the reader is referred to for full technical details [13]. Briefly, the EEG was recorded using a DC-coupled, TMS-compatible amplifier (Actichamp, BrainProducts, GmbH). Signals were recorded with a sampling rate of 5000 Hz from 63 active electrodes mounted on a cap (actiCAP) in accordance with the international 10–10 system. The pre-processing pipeline included epoching (−1 to 1 s), artefact removal by ICA, and filtering (band stop 48–52 Hz, band pass 1–100 Hz). The procedures described in the original research were performed in accordance with the Declaration of Helsinki and approved by the human subjects review board of the University College London. Participants gave written informed consent prior to the experimental session. Each dataset was composed of 120 epochs, and three experimental conditions were considered. Condition 1 (C1) corresponded to TEPs obtained during masking of the TMS click; in condition 2 (C2), only AEPs were present; in condition 3 (C3), effective cortical stimulation was obtained without masking of the TMS click, so as to obtain mixed responses due to both direct cortical stimulation and auditory input. Signals were divided into three time windows of interest (ToI). These corresponded to the main peaks of the global mean field potential (GMFP) and were named early (15–65 ms), middle (66–120 ms), and late (121–270 ms) [13]. Our main aim was to investigate the classification accuracy (CA) of our neural network in the middle ToI, where a cluster-corrected permutation approach failed to disclose significant differences between C1 and C2 [13]. However, we extended our analysis to all ToIs to observe the classification performance of the algorithm when clearer differences were present between conditions (early and late ToIs). Due to the large number of observations required, single trial data were initially used for the training phase of the network in most experiments (except for B3, as detailed below).

In order to measure the generalization ability of the neural network, i.e., the CA on new input–output pairs, the available data were divided into a training set and test set; the former was used for the training phase, while the purpose of the latter was to measure the CA, defined as the ratio between the number of correctly classified samples and the whole number of samples of the test set. The neural network was trained by using the built-in MATLAB function *trainNetwork*. As an optimization algorithm to train the network, we chose *adam* [25], which is a popular stochastic method that uses random subsamples at each iteration and employs estimates of the 1st moment and the 2nd raw moment of the gradient. We set the maximum number of iterations to 100. In each run, the data of the training set were first normalized by subtracting the mean and dividing by the standard deviation. We used a single fully connected layer with three neurons. As output layers, we used a softmax layer, which applies a softmax function to the input, followed by a classification layer. The latter is needed because the neural network is used for classification purposes, while the softmax layer was chosen as it is known to be useful for computing probability estimates in the context of multiclass problems.

**Figure 2:**
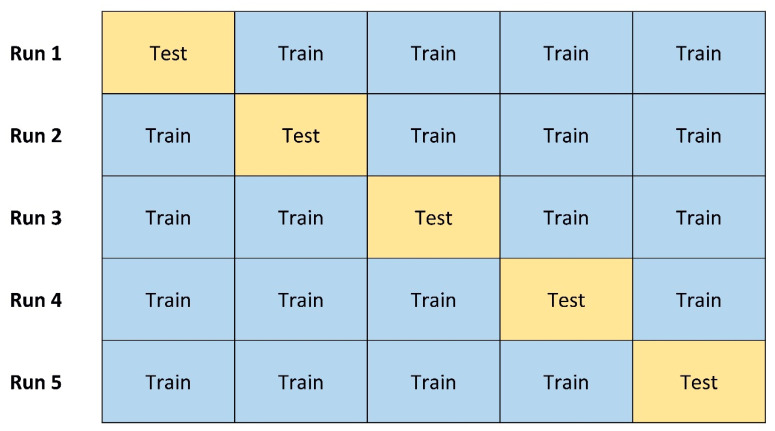
Example of 5-fold cross validation (see text for details).

The aim of our numerical experiments was to assess the ability of neural networks to correctly identify the contribution of AEPs to the TEP by discriminating signals obtained in different conditions (C1, C2, C3), as explained above. We applied a *k*-fold cross validation, with *k* being a positive integer number (5, in our case) [26,27]; this technique consists of dividing the dataset into *k* subsets of (approximately) equal size, such that one of them is used as the test set and the remaining ones form the training set. This is repeated *k* times, in order to use a different subset as the test at every run. The classification error is then computed as the average over the *k* test sets (Figure 2).

**Figure 3:**
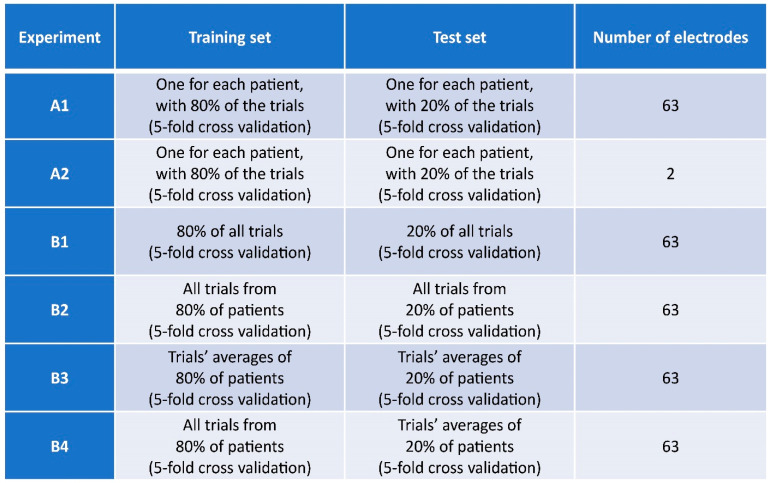
Structure of the different numerical experiments (see Materials and Methods section for details).

The experiments were divided into two groups, as explained in Figure 3.

**Figure 4:**
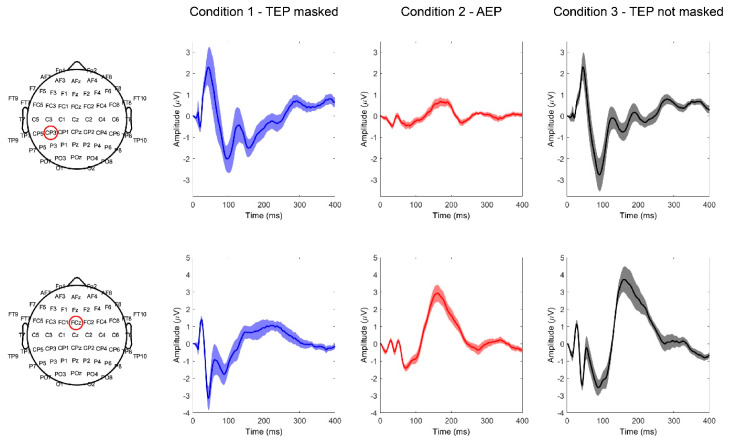
Average TEPs recorded from electrode CP3 (upper row) and FCz (lower row) in the three different stimulation conditions (C1−TEP masked; C2−AEP; C3−TEP not masked).

In group A, we aimed to investigate the ability of the network to correctly classify TEPs at the single-subject level. This was done by using either the whole set of 63 electrodes (A1) or the 2 electrodes (CP3 and FCz, according to the 10–20 International System of Electrode Placement) where signals showed maximum differences between conditions C1 and C2, due to the different topographies of TEPs and AEPs [13] (Figure 4).

**Figure 5:**
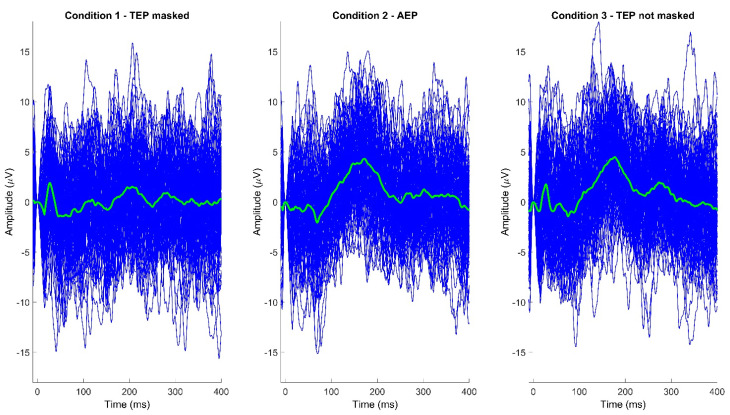
Example of single-trial and average TEPs, from a single subject, recorded from electrode Cz in the three different stimulation conditions (C1−TEP masked; C2−AEP; C3−TEP not masked).

In this set of calculations, 80% of the trials were used both for training and test sets. Group B experiments were designed to investigate whether a correct classification between conditions could be reached if data from all subjects were pooled together. In experiment B1, we trained the classifier using 80% of all trials, while the remaining 20% were used as a test set; for each participant, trials were mutually assigned to training or test sets. However, with the aim of building classifiers suited for practical usage, in experiments B2, B3, and B4, training trials and test trials were obtained from different subsets of subjects. In particular, 80% of participants was used to obtain training data and 20% for test data. The nature of the variables considered was different in the last three experiments. Whereas single trials were used for both training and test data in B2, the average over all trials for each subject (see Figure 5) was used for both phases in experiment B3. This was done to investigate whether signal averages, which are more commonly used for TMS-EEG studies, could be suitable in this context. In the last experiment B4, single-trial data were used for training, while averages were used as test sets, to investigate whether features of single epochs were sufficient to correctly classify average TEPs (Figure 5).

Since, in most experiments, only one value of the CA was computed under each condition, there were no distributions to allow for a formal statistical analysis; therefore, a descriptive account of the results is given below.

## 3. Results

**Figure 6:**
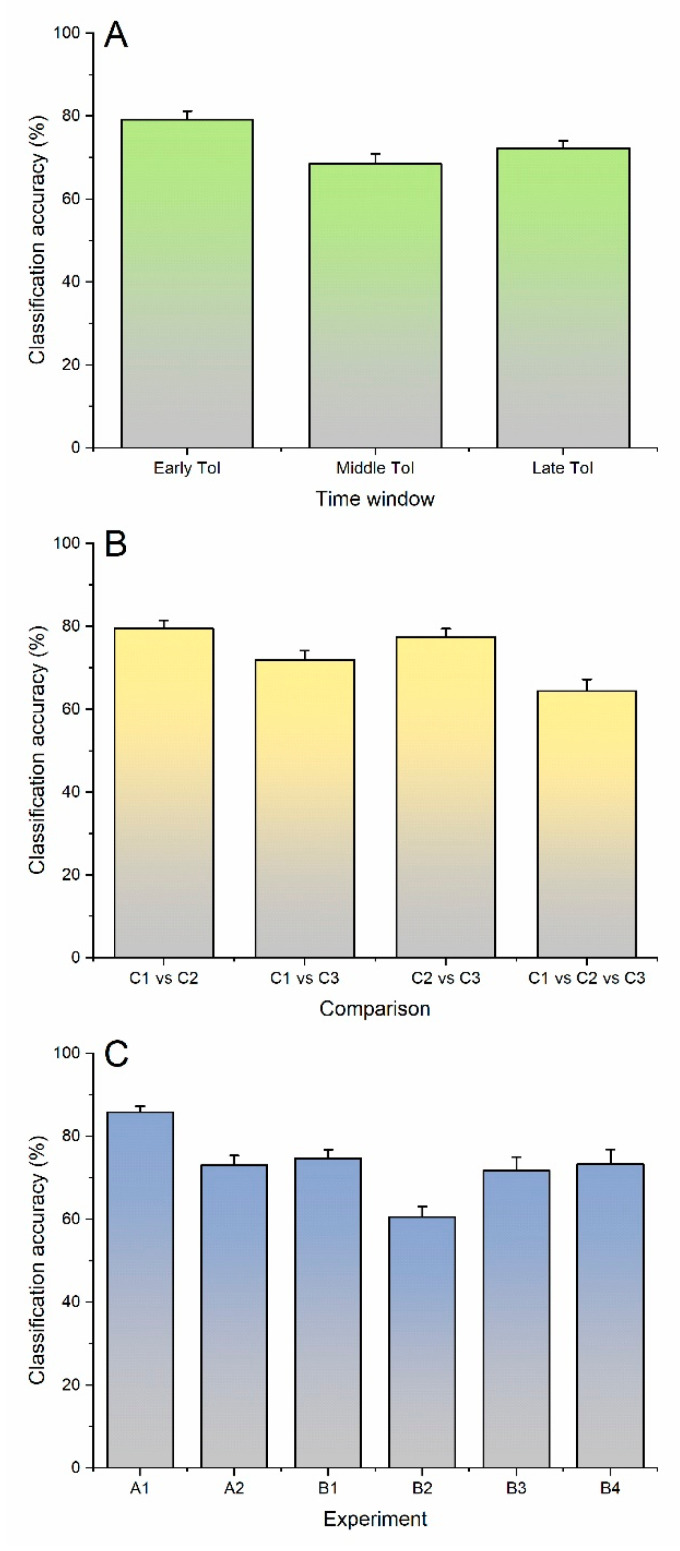
Trends of classification accuracy in the different ToIs (**A**), stimulation conditions (**B**), and numerical experiments (**C**).

A full account of CA values is given in Table 2. Figure 6 depicts the average CA separately for ToIs, comparisons between stimulation conditions, and experiments.

As a general trend, results were better in the early ToI, intermediate in the late ToI, and worse in the middle ToI (Figure 6A). In terms of stimulation conditions, C1 and C2 were the most effectively discriminated, followed by C2 vs. C3 and C1 vs. C3. Bivariate comparisons generally yielded a higher accuracy than the comparison of all three conditions together (Figure 6B); this is not surprising, as, in general, one expects a lower accuracy when passing from binary to multiclass classification.

CA values for different experiments are indicated in Figure 6C. Experiment A1, which was conducted to classify the trials at the single subject level, considering data from all 63 recording electrodes, resulted in a higher CA; the latter was lower when only two electrodes (CP3 and FCz) were used (experiment A2).

Differently from experiments A1 and A2, which were carried out at the single subject level, group B experiments were performed at the group level. In experiment B1, where trials from all subjects were pooled, the CA was lower than in A1. The CA in experiment B2, where separate subgroups of patients were used for training and test phases, was generally worse than in B1. Compared to B2, accuracy values were higher in experiment B3, where signal averages were used for both the training and test phases. Importantly, the results were similar to B3 if single trials were used for classification and averages for the test phase (experiment B4).

## 4. Discussion

In this proof-of-concept work, we investigated the ability of a machine learning algorithm to classify AEPs and TEPs obtained in different noise masking conditions. Overall, CA was higher in the early ToI, lower at the group level, when different subjects were used for training and test phases, and again lower when three stimulation conditions instead of two were compared. The CA was also higher when signal averages, rather than single-trial TEPs, were used.

### 4.1. Classification Accuracy in Different Time Windows of Interest

The main objective of our study was to discriminate TEPs from AEPs in a time window (the middle ToI of the present work) where separation with common statistical procedures was proven to be difficult [13]. Therefore, it is not surprising that CA values were lowest in the middle ToI and higher in the late ToI, where, conversely, the presence of a central P200 in the AEPs but not in the TEPs makes the distinction between the two easier [13]. Two results were, however, unexpected. First, the CA was high (84.94%) also when comparing the late ToI in C2 and C3, which share auditory input. Second, the highest CA was found in the early ToI. In particular, a very high (92.29%) CA in the early ToI was found not only when comparing masked TEPs and AEPs (C1 and C2), which show clear differences, but also when comparing masked and non-masked TEPs (90.07%) (C1 and C3), which appear very similar (Table 2 and Figure 6). While it is difficult to interpret the latter result, it is possible that the machine learning algorithm used was particularly sensitive in detecting differences related to high-frequency, early- and middle-latency auditory components, which occur within 50 ms after the stimulus [28] and are, therefore, present in the early ToI. These results point to the ability of our algorithm to highlight differences between TEP conditions that are not easily observed or disclosed by permutation-based statistics.

### 4.2. Classification Accuracy in Different Conditions of Transcranial Stimulation and Noise Masking

The CA in different stimulation conditions yielded the expected results (Figure 6B). The highest average value was obtained when comparing C1 and C2, i.e., masked TEPs and AEPs, which present marked differences due to auditory input suppression (C1) and the absence of direct cortical stimulation (C2). A slightly lower CA was reached comparing C2 and C3 (AEP and non-masked TEPs), which differed only in terms of direct cortical activation by TMS. The comparison between C1 and C3, which shared similar transcranial activation but differed in terms of AEPs suppression, yielded lower CA (around 70%). The simultaneous comparison of all three conditions was the one providing the lowest CA (around 64%), probably due to the increased complexity of the design.

### 4.3. Classification Accuracy in Different Experiments

Average CA values in different experiments are described in Figure 6C. Experiment A1, where the whole set of 63 electrodes was used to classify TEPs at a within-subject level, yielded the best results, with an average CA of more than 85%. This figure is particularly noteworthy considering that TEPs are usually quantified by averaging tens or hundreds of trials, due to a low signal-to-noise ratio (SNR) [15]. This suggests that the different cortical activation pattern caused by transcranial activation and auditory input has recognizable features even in low SNR conditions. In experiment A2, we tested the hypothesis that the CA would be increased in conditions similar to A1, but instead using signals from the two electrodes showing maximal differences between C1 (CP3, masked TEPs) and C2 (FCz, AEPs). The result was the opposite, i.e., the CA was sensibly lower than in experiment A1 (~73%). These results might indicate that even signals from electrodes that are not intuitively linked to differences between conditions contain useful information for classification.

In a further set of experiments, we tested the performance of our classifier at the group level. In experiment B1, where data from each subject were used for both training and test sets, the CA (~74%) was lower than in A1. Average CA values further decreased (~60%) in experiment B2, where data from two independent subject samples were used for training and test phases. It is likely that the CA in both experiment B1 and B2 suffered from the noisy nature of the single-trial TMS-EEG data. In B1, the CA was probably higher than in B2 due to the fact that inter-subject variability was the same for the data used in the training and test phases, whereas in the latter condition, the independence of the data used in the two phases accounted for a decrease in common features. It is very important to note that, in similar randomization conditions, the CA was increased (~72%) when average TEPs were used; this indicates that, in this context, a cleaner signal more than compensated for the much lower number of observations used in the training phase. It is unclear, however, whether this positive effect was due to the use of average TEPs in the training or test phase. Further information on this point was provided by experiment B4, where a CA (~73%) similar to that in experiment B3 was obtained. This indicates that the efficacy of the training phase was stable when a larger amount of more noisy data was used, provided that the test set still included average TEPs.

## 5. Conclusions

This proof-of-concept study demonstrates the feasibility of using machine learning algorithms to address the contamination of TEPs by AEPs, an important methodological problem in TMS-EEG literature [11,12,13]. Some results were predictable, such as the average CA when comparing different conditions in terms of AEP suppression (Figure 6B). Other results, such as the very high CA in the early ToI (Figure 6A), were less intuitive. Our study provides novel insights into the contamination of TEPs by AEPs, revealing both expected and unexpected patterns in the data. These findings suggest that machine learning algorithms may offer advantages over traditional statistical approaches.

Although results were very positive at the single-subject level when comparing two stimulation conditions, the ability of the tested classifier was slightly lower when classification was attempted on all three stimulation conditions and when including data from different subjects in the training and test phases. While our results are encouraging, there are several limitations to this study, including the use of time-domain signals only and the relatively small sample size.

In conclusion, machine learning algorithms represent a promising solution to objectively assess the contamination of TEPs by AEPs. However, the quality of the results obtained by a machine learning approach can strongly depend on the number of available samples. Future studies should explore the use of different measures, such as metrics in the time–frequency domain, and larger datasets, both within and across laboratories, to further validate the potential of machine learning algorithms to objectively assess contamination of TEPs by AEPs. Our findings have important implications for the design and interpretation of TMS-EEG studies and may ultimately help to improve the accuracy and reproducibility of this valuable technique in neuroscience research.

## Figures and Tables

**Table 1:** Features of the two different N100 waves elicited either by the TMS click (“Auditory” N100) or resulting from direct cortical activation by transcranial magnetic stimulation (“TEP” N100). See Introduction for details.

«Auditory» N100	«TEP» N100
Can be suppressed by noise masking/ear defenders	Cannot be suppressed by noise masking/ear defenders
Located at the vertex	Located at the stimulation site
Followed by a P200	Not followed by a P200
Shorter latency	Longer latency

**Table 2:** Results of all the numerical experiments. Percentages refer to the classification accuracy of the neural network in each comparison.

	Early ToI				Middle ToI				Late ToI			
	C1 vs. C2	C1 vs. C3	C2 vs. C3	C1 vs. C2 vs. C3	C1 vs. C2	C1 vs. C3	C2 vs. C3	C1 vs. C2 vs. C3	C1 vs. C2	C1 vs. C3	C2 vs. C3	C1 vs. C2 vs. C3
A1	92.29%	90.07%	91.69%	86.83%	87.84%	86.34%	88.44%	81.17%	83.65%	81.80%	84.94%	75.74%
A2	85.28%	73.43%	82.65%	69.26%	80.18%	66.16%	78.23%	61.22%	76.63%	68.56%	75.01%	60.08%
B1	84.24%	77.24%	84.40%	71.65%	75.21%	70.53%	77.57%	62.24%	76.49%	76.46%	77.53%	64.84%
B2	72.27%	61.44%	74.12%	56.05%	62.97%	54.25%	62.65%	43.73%	67.54%	61.45%	62.59%	48.62%
B3	91.67%	66.67%	84.17%	72.78%	75.83%	65.83%	63.33%	50.00%	79.17%	79.17%	68.33%	65.00%
B4	89.17%	79.17%	87.50%	75.55%	65.83%	60.00%	75.83%	47.22%	84.17%	75.83%	73.33%	67.22%

## Data Availability

The data and code presented in this study are available upon request from the corresponding author.
